# 

**Published:** 1850-01-01

**Authors:** 


### CONTENTS.

PAGE

Address to our Readers . . . . . . . . . . . . . iii
On Hypŏchondriasis . . . . . . . . . . . . . . 1
The Law of Lunacy . . . . . . . . . . . . . . . 14
On Suicide—Verdicts of Felo-de-se . . . . . . . . . 19
Politics and Insanity . . . . . . . . . . . . . . 31
Autobiography of the Insane . . . . . . . . . . . 40
On Cretinism . . . . . . . . . . . . . . . . 54
Capital Punishments, psychologically considered . . . . . 61
British Lunatic Asylums . . . . . . . . . . . . 76
Analysis of Crime . . . . . . . . . . . . . . 94
Bentley's Miscellany and Mad Doctors . . . . . . . . 123
Correspondence from Paris . . . . . . . . . . . . 126
Miscellaneous Notices . . . . . . . . . . . . . 137
Congratulatory Address to Samuel Gaskell, Esq. . . . . 139

